# Meta-analysis of the gut microbiota in predicting response to cancer immunotherapy in metastatic melanoma

**DOI:** 10.1172/jci.insight.140940

**Published:** 2020-12-03

**Authors:** Angelo Limeta, Boyang Ji, Max Levin, Francesco Gatto, Jens Nielsen

**Affiliations:** 1 Department of Biology and Biological Engineering, Chalmers University of Technology, Gothenburg, Sweden.; 2 Novo Nordisk Foundation Center for Biosustainability, Technical University of Denmark, Lyngby, Denmark.; 3 Wallenberg Laboratory for Cardiovascular Research, Department of Molecular and Clinical Medicine, and; 4 Department of Oncology, Institute of Clinical Sciences, Sahlgrenska Academy, University of Gothenburg, Gothenburg, Sweden.; 5 Department of Oncology, Sahlgrenska University Hospital, Gothenburg, Sweden.; 6 Elypta AB, Stockholm, Sweden.; 7 BioInnovation Institute, Copenhagen, Denmark.

**Keywords:** Microbiology, Oncology, Bioinformatics, Cancer immunotherapy, Melanoma

## Abstract

**BACKGROUND:**

Identifying factors conferring responses to therapy in cancer is critical to select the best treatment for patients. For immune checkpoint inhibition (ICI) therapy, mounting evidence suggests that the gut microbiome can determine patient treatment outcomes. However, the extent to which gut microbial features are applicable across different patient cohorts has not been extensively explored.

**METHODS:**

We performed a meta-analysis of 4 published shotgun metagenomic studies (*N_tot_* = 130 patients) investigating differential microbiome composition and imputed metabolic function between responders and nonresponders to ICI.

**RESULTS:**

Our analysis identified both known microbial features enriched in responders, such as *Faecalibacterium* as the prevailing taxa, as well as additional features, including overrepresentation of *Barnesiella intestinihominis* and the components of vitamin B metabolism. A classifier designed to predict responders based on these features identified responders in an independent cohort of 27 patients with the area under the receiver operating characteristic curve of 0.625 (95% CI: 0.348–0.899) and was predictive of prognosis (HR = 0.35, *P* = 0.081).

**CONCLUSION:**

These results suggest the existence of a fecal microbiome signature inherent across responders that may be exploited for diagnostic or therapeutic purposes.

**FUNDING:**

This work was funded by the Knut and Alice Wallenberg Foundation, BioGaia AB, and Cancerfonden.

## Abstract 2

A meta-analysis of studies investigating the impact of the gut microbiome on response to cancer immunotherapy in patients with metastatic melanoma

## Graphical abstract

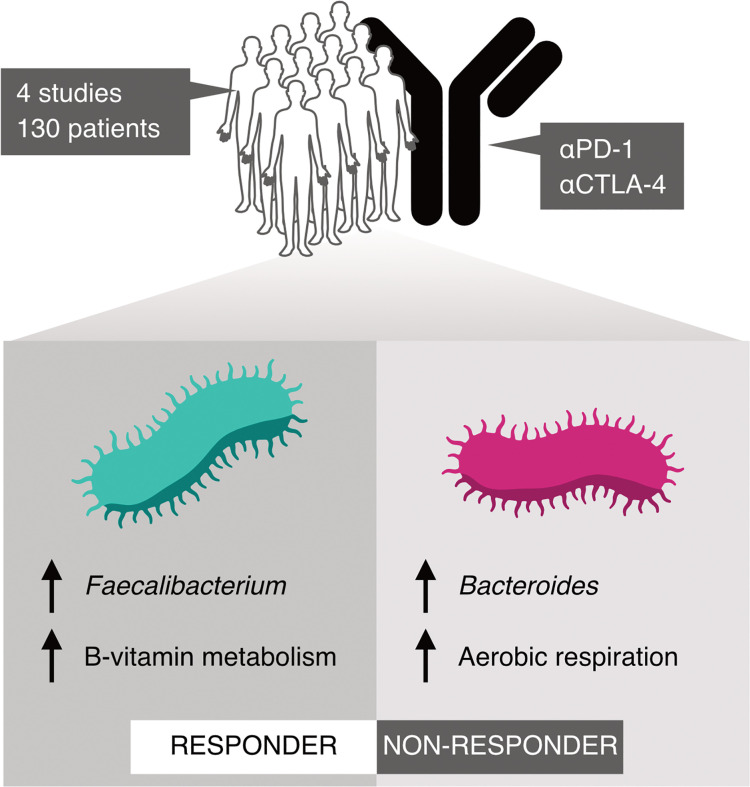

## Introduction

Immunotherapy with immune checkpoint inhibitors (ICIs) has radically improved survival across a range of cancers in the last decade (1). ICIs are antibodies blocking the interaction between negative costimulatory receptors present on the cell surface of certain immune cells, such as cytotoxic T lymphocytes (CD8^+^ T cells), and their ligands (2). Several cancers actively suppress these receptors to silence or exhaust surrounding immune cells, thereby evading immune surveillance and subsequent cytolysis. Blocking this interaction serves as a way to keep T cells active, allowing a sustained destruction of cancer cells by the immune system. ICIs targeting the programmed cell death 1 protein (PD-1) and programmed death ligand 1 (PD-L1), alone or in combination with the cytotoxic T lymphocyte–associated antigen 4 (CTLA-4), are now used as first-line therapy for late-stage cancers, including metastatic melanoma (MM), non–small cell lung cancer (NSCLC), and renal cell carcinoma (RCC). In fact, 5-year overall survival rates for patients with MM soared from 16% to 52% after the introduction of an anti–CTLA-4 + anti–PD-1 combination therapy (3, 4).

Nonetheless, half the patients do not respond to ICI therapy, and predictions of which patients belong to the responder (R) or nonresponder (NR) phenotype are not yet possible. Previous studies examining the intrinsic oncogenic features of the tumor have revealed several processes that negatively correlate with treatment response; see ref. 5 for a comprehensive review on the topic. These include low mutational burden (6), presence of mutations in the interferon-γ (IFN-γ) signaling pathway (7), downregulation of the human leukocyte antigen genes (8), and the presence of a transcriptional signature characterized in part by overexpression of cyclin-dependent kinase activity (9).

ICI therapy does not target the cancer cells directly but rather their interplay with immune cells. Therefore, it is important to consider the role of whole-body immunity in each patient. Whole-body immunity is affected through several host and lifestyle factors, such as age, diet, and environment. It has recently emerged that the complex interplay between lifestyle and host immunity is in part mediated by the composition of the gut microbiota. Microorganisms in our gut are continually interacting with the gut epithelial barrier, through direct interactions with the microorganisms themselves, or through the myriad of diverse compounds they produce when metabolizing our diet (10, 11). Immune cells lining the gut mucosa have the privileged position to sense and be affected by changes in microbial ecology, subsequently triggering the activity of near and distal T cells, which can ultimately affect ICI outcomes (12). Previous studies in mice revealed that germ-free or antibiotic-treated mice were unable to respond to ICI therapy (13, 14). Treatment outcomes for sarcoma and melanoma mouse models could then be restored by introducing specific bacterial taxa from the Bacteroides and Bifidobacterium phyla, respectively (13, 14). The establishment of a causal relationship between specific taxa and their ability to significantly affect ICI outcomes has also been extended to humans. However, the exact mechanisms and requirements remain unclear. Because the gut microbiome composition varies tremendously across people, it remains difficult to know how well one can extend these findings across different regional boundaries. In fact, each study reports a unique set of microbial patterns predictive of response to ICI therapy. This issue could potentially hamper the development and applicability of pre- and probiotics for boosting ICI outcomes across the population.

In this study, we collected the published whole metagenome shotgun sequencing (MGS) gut microbiome data sets obtained from patients with MM undergoing anti–PD-1, –PD-L1, –CTLA-4, or combination immunotherapy (15–18) and performed a meta-analysis on the compositional and functional features of the microbiota between R versus NR across studies.

## Results

### Study selection and characteristics.

After a manual search from literature databases, we included any studies investigating the gut microbiome effects of cancer ICI therapy with publicly available fecal MGS data and associated metadata (Table 1, Methods). We excluded nonfecal samples from each data set, along with any samples taken during treatment. In order to categorize each patient as belonging to either a responder or nonresponder phenotype, we sourced the available metadata in each study. Treatment responses were evaluated through the Response Evaluation Criteria in Solid Tumors 1.1 (RECIST 1.1) as determined in the original study at first response evaluation (19). Patients with complete or partial response, according to RECIST 1.1 criteria, were classified as responders, whereas patients with stable or progressive disease were classified as nonresponders. We initially included a study investigating links between microbiome composition and response to immunotherapy in RCC and NSCLC patients (20). However, we failed to identify any remarkable shifts in microbiome composition according to our predefined response criteria, both in the study itself and when pooling the data across studies (Supplemental Figures 1–4 and Supplemental Figure 6; supplemental material available online with this article; https://doi.org/10.1172/jci.insight.140940DS1). For consistency, we decided to limit our scope to the MM subgroup of patients. This was further motivated by the fact that the Peters et al. data set (18), which we used for validation, consists exclusively of patients with MM. In total, the final set of exclusively MM patients used throughout this analysis included 66 responders and 64 nonresponders.

### Compositional differences of the microbiome between responders and nonresponders.

**Figure 1:**
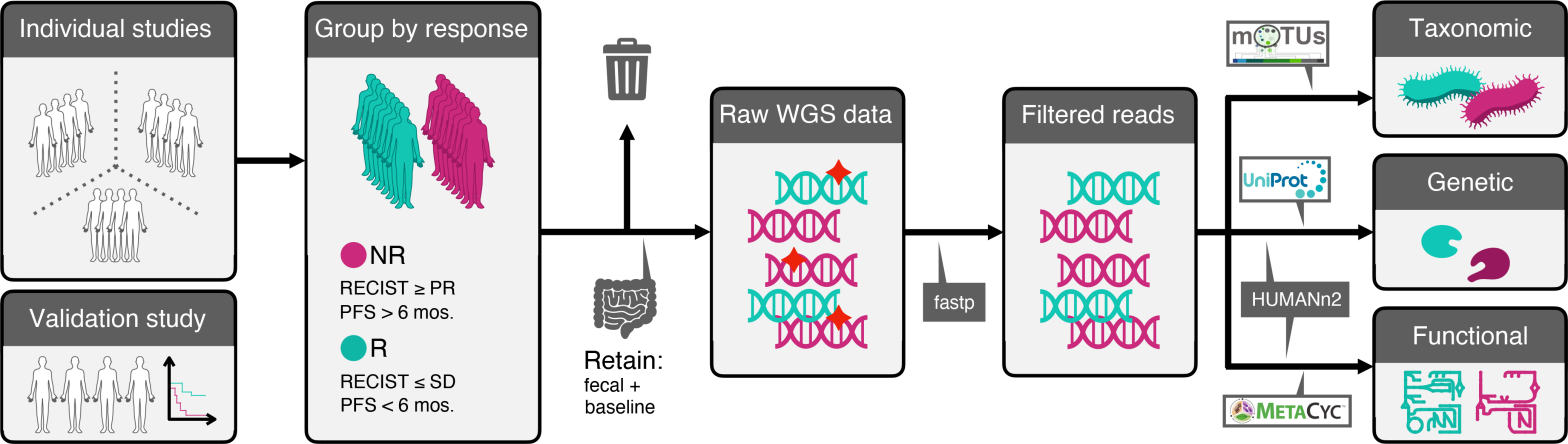
Meta-analysis workflow. In brief, fecal MGS data at baseline from 4 studies (*N* = 130) comparing differences in microbiome composition between R and NR were systematically reanalyzed at the taxonomic, genetic, and functional level. We set aside data from one of the studies (18) (*N* = 27) in order to validate our findings in an independent cohort. WGS, whole-genome sequencing.

All the raw sequencing data and metadata for each study were consistently reprocessed and reanalyzed according to a self-developed bioinformatics pipeline as described in the Methods section (Figure 1). Examining taxonomic distribution at the family and genus level did not reveal remarkable differences between NR and R groups (Supplemental Figure 1). When grouped by study, we identified larger relative abundances of taxa belonging to the Lachnospiraceae and Ruminococcaceae families in the Frankel et al. data set. At the genus level, the Frankel et al. data set tended to contain increased relative abundances of the *Blautia* genus compared with the remaining studies. Comparisons of alpha-diversity, a measure of microbial community diversity within each patient, at the species level between response groups for the pooled data did not result in significant differences (*P* > 0.05, Wilcoxon’s rank-sum test) according to multiple alpha-diversity indices. The lack of significant differences in diversity between R and NR hints that a few individual microbes might drive phenotypic differences in these patients.

**Figure 2:**
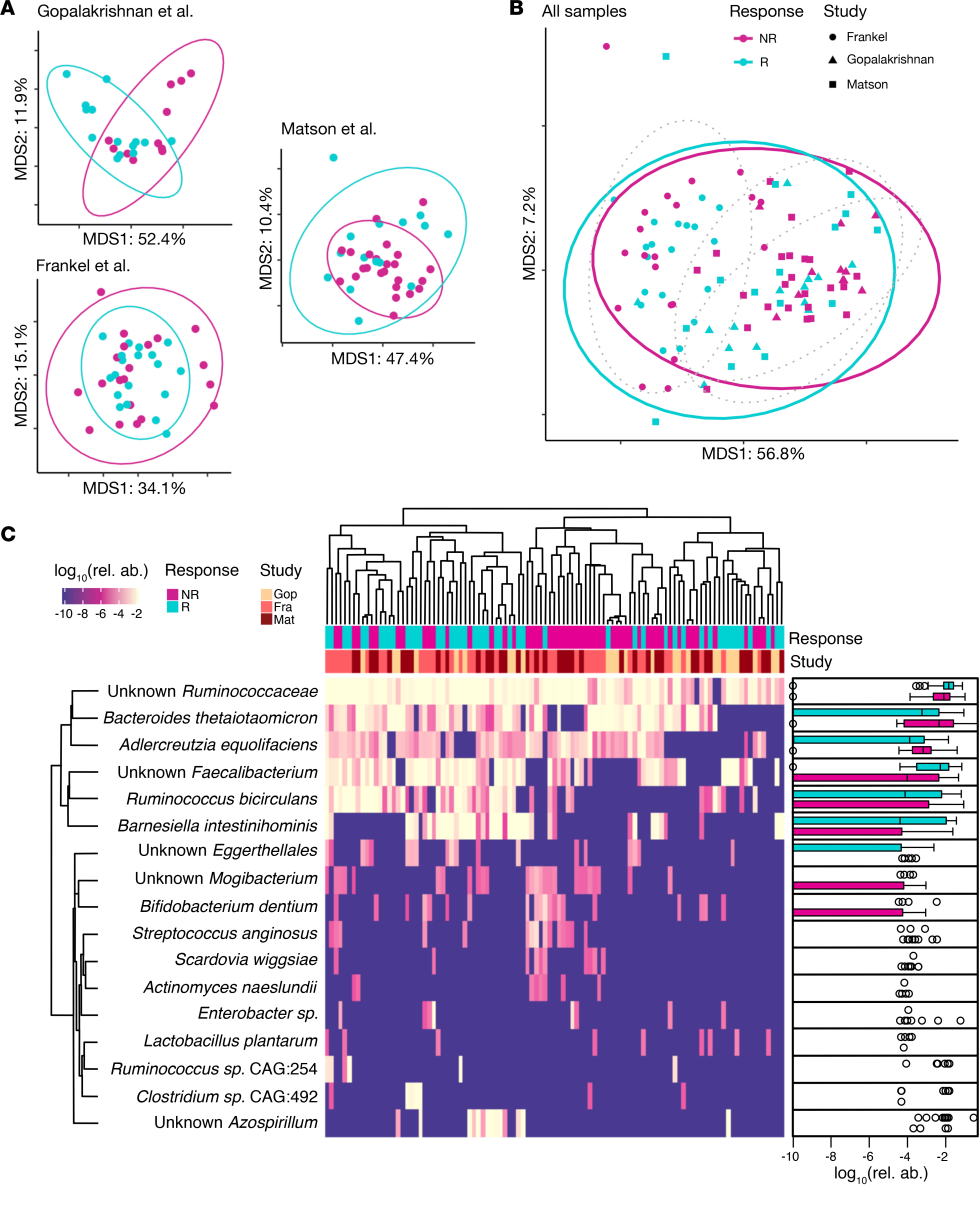
Differences in microbial abundances between responders and nonresponders to ICI therapy. (**A**) Multidimensional scaling (MDS) analysis using weighted UniFrac distances of samples (*N* = 103) pertaining to each study and grouped by response. (**B**) MDS plot of all samples included in the meta-analysis, grouped by response. Dashed and solid lines indicate 95% normal confidence ellipses for each study and response group, respectively. (**C**) Hierarchical clustering of log-normalized abundances of differentially abundant taxa between R and NR for only the melanoma subset of patients. Seventeen operational taxonomic unit (OTUs) were identified as differentially abundant with *P* < 0.05 (Wilcoxon’s rank-sum test).

We next performed beta-diversity analysis, which measures microbial community diversity between patients, based on taxonomic composition at the species level using multidimensional scaling (MDS) with weighted UniFrac distances, to see if we could cluster patients in R and NR separately. For each individual study, we only observed a marked difference in beta-diversity between the R and NR groups in the Gopalakrishnan et al. data set (15) (Figure 2A). Pooling the data and running MDS did not produce any apparent difference in beta-diversity between R and NR patients; in fact samples from individual studies tended instead to cluster together (Figure 2B).

Given that no consistent difference between R and NR could be observed at the global level, we investigated to extract differentially abundant species between R and NR using several methods (Methods, Supplemental Figure 4). Differential abundance testing between R and NR for the melanoma subset yielded 17 differentially abundant species (*P* < 0.05, unadjusted, Wilcoxon’s rank-sum test, Supplemental Table 1). Hierarchical clustering of patients using only the differentially abundant species revealed 2 clusters, one enriched in R and the other enriched with NR (Figure 2C, *P* = 0.0013, Fisher’s exact test). Among the most abundant species, we observed that R patients were enriched in unknown *Ruminococcaceae* species, unknown *Faecalibacterium* species, *Ruminococcus bicirculans*, and *Barnesiella intestinihominis*, whereas NR patients were enriched in *Bacteroides thetaiotaomicron*, *Adlercreutzia equolifaciens*, *Bifidobacterium dentium*, and unknown *Mogibacterium*. Notably, we did not observe any major clustering of samples by study of origin using the differentially abundant species. To check the validity of our approach, we reanalyzed differentially abundant taxa between R and NR on a per-study basis (Supplemental Figure 3). Consistent with our pooled findings, we replicated the majority of the findings in each individual study (some taxa identified to be differentially abundant in the original study but not in the present meta-analysis all had unadjusted *P* values slightly higher than 0.05). This indicates that the gut microbiome is heterogenous between patient populations, but there seems to exist a common species repertoire across the majority of patients.

In order to identify whether there were any differences in the topology of the microbial association network between the R and NR groups, as well as to map out microbes serving as network hubs, we performed a co-occurrence analysis at the genus level on the pooled data (Supplemental Figure 5). Highly interconnected microbes in the co-occurrence networks can serve as regulators for the general community structure and might be useful for targeted therapeutics, to alter the microbiome structure from an NR to R phenotype. We observed similar network interconnectivity in R versus NR. However, whereas *Mogibacterium*, *Anaerococcus*, and *Eggerthella* formed the largest hubs in the R network, in NR, the largest hubs were dominated by *Subdoligranulum*, *Lachnoclostridium*, *Eggerthella*, and *Streptococcus*. While *Mogibacterium* was identified as being increasingly abundant in NR, the fact that it co-occurs with several bacterial species in the R network suggests that it might have an interesting role for maintaining an R microbiome structure. Nonetheless, it is important to note that co-occurrence does not directly imply any causal dependence between microbes, e.g., metabolite exchange as a form of symbiosis.

### Functional differences of the microbiome between responders and nonresponders.

**Figure 3:**
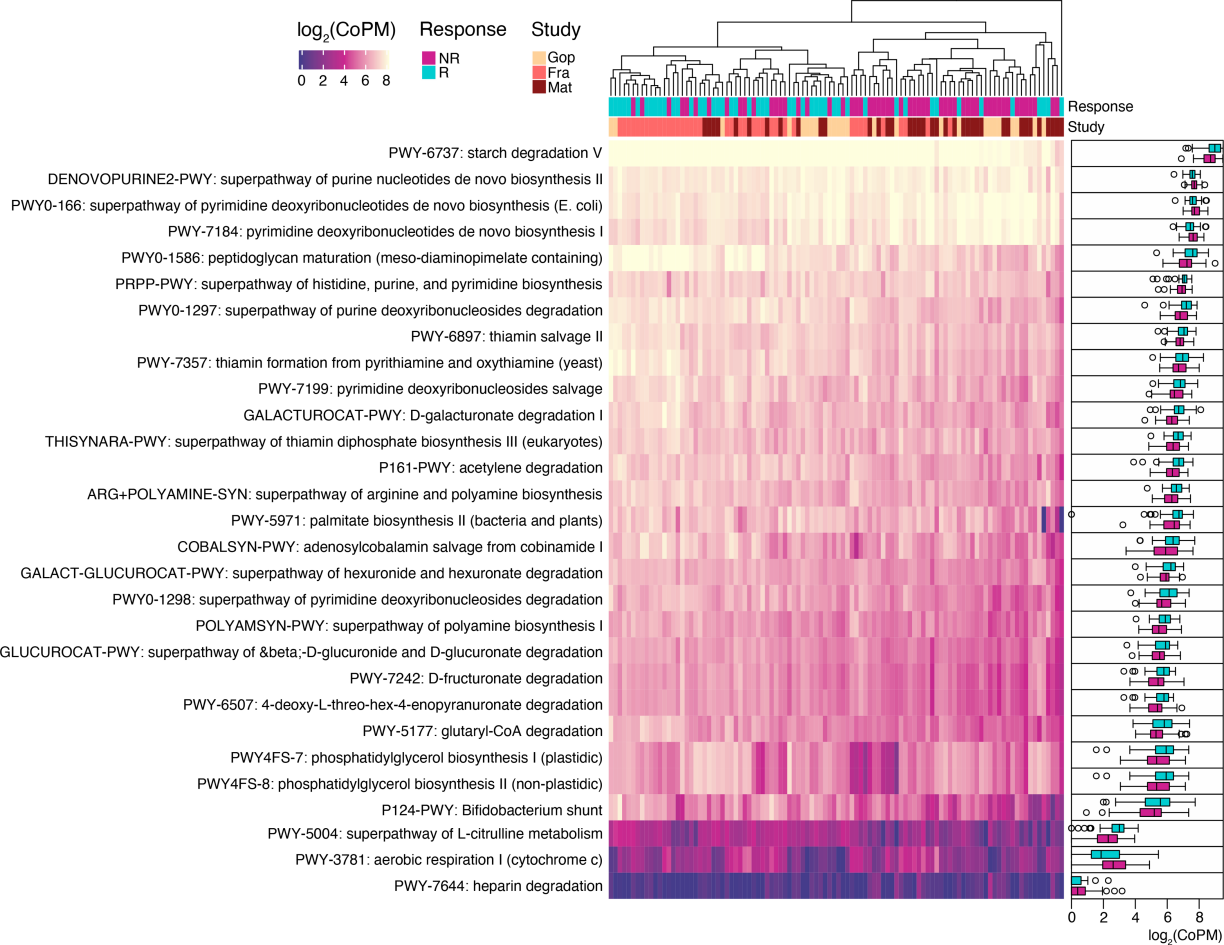
Alterations in the functional potential of the gut microbiome between responders and nonresponders to ICI therapy. Log-normalized abundances reported as copies per million (CoPM, analogous to TPM for RNA-Seq data) of differentially abundant MetaCyc pathways present in the fecal microbiome of patients (*N* = 103). Twenty-nine pathways were identified as differentially abundant with *P* < 0.05 (unadjusted, Wilcoxon’s rank-sum test). PWY-tags preceding each pathway name are unique IDs associated with each metabolic pathway on MetaCyc. Hierarchical clustering dendrogram for the pathways is omitted due to figure size constraints.

In order to understand the functional consequences of differential abundance of specific species in R versus NR, we next focused on genes associated with metabolic pathways. We initially examined the genetic content on a gene-by-gene basis; however, we did not find anything remarkable. To enhance interpretability of the findings, we examined genes mapped onto MetaCyc pathways, which resulted in approximately 500 features. Dimensionality reduction analysis of the functional content between R and NR showed minimal separation between each of the groups when pooling all the studies (Supplemental Figure 6). Differential abundance tests of each MetaCyc pathway — defined as the lowest number of reads mapping to individual pathway-associated genes — between R and NR resulted in 29 differentially abundant pathways (Supplemental Table 1, P** < 0.05, unadjusted, Wilcoxon’s rank-sum test). Hierarchical clustering of patients using these 29 pathways resulted in the emergence of 2 large clusters enriched either in R or in NR (Figure 3, P** < 0.0001, Fisher’s exact test). The majority of these differentially abundant pathways were enriched in R, covering a wide array of biological processes, including starch and glycoside degradation, thiamine metabolism, cobalamin metabolism, peptidoglycan maturation, and nucleotide degradation. The differentially abundant pathways enriched in NR covered a narrower span of processes, specifically nucleotide biosynthesis and aerobic respiration, although the latter was represented in low copies across samples. We were interested in manually examining the reads mapping onto the aerobic respiration pathway in order to identify potential pathogens. Although a large part remained unmapped, the annotated reads were found to be belonging to *Campylobacter* and *Citrobacter* genera, both of which are members of the Proteobacteria phylum.

Reanalyzing the functional content of each study individually, we again managed to replicate many of the original findings in the original studies (Supplemental Figure 7). These included upregulation of inositol metabolism in R, reported by Frankel et al. (17), and degradation pathways enriched in R and biosynthesis pathways enriched in NR, reported by Gopalakrishnan et al. (15). Matson et al. did not report any functional analysis of their MGS data (16), but we managed to observe increased nucleotide biosynthesis in NR and increased biosynthesis of more complex organic compounds, such as isoprenoids, polyamines, and coenzymes, in R.

### Translating microbiome signatures predictive of response to ICI across studies.

**Figure 4:**
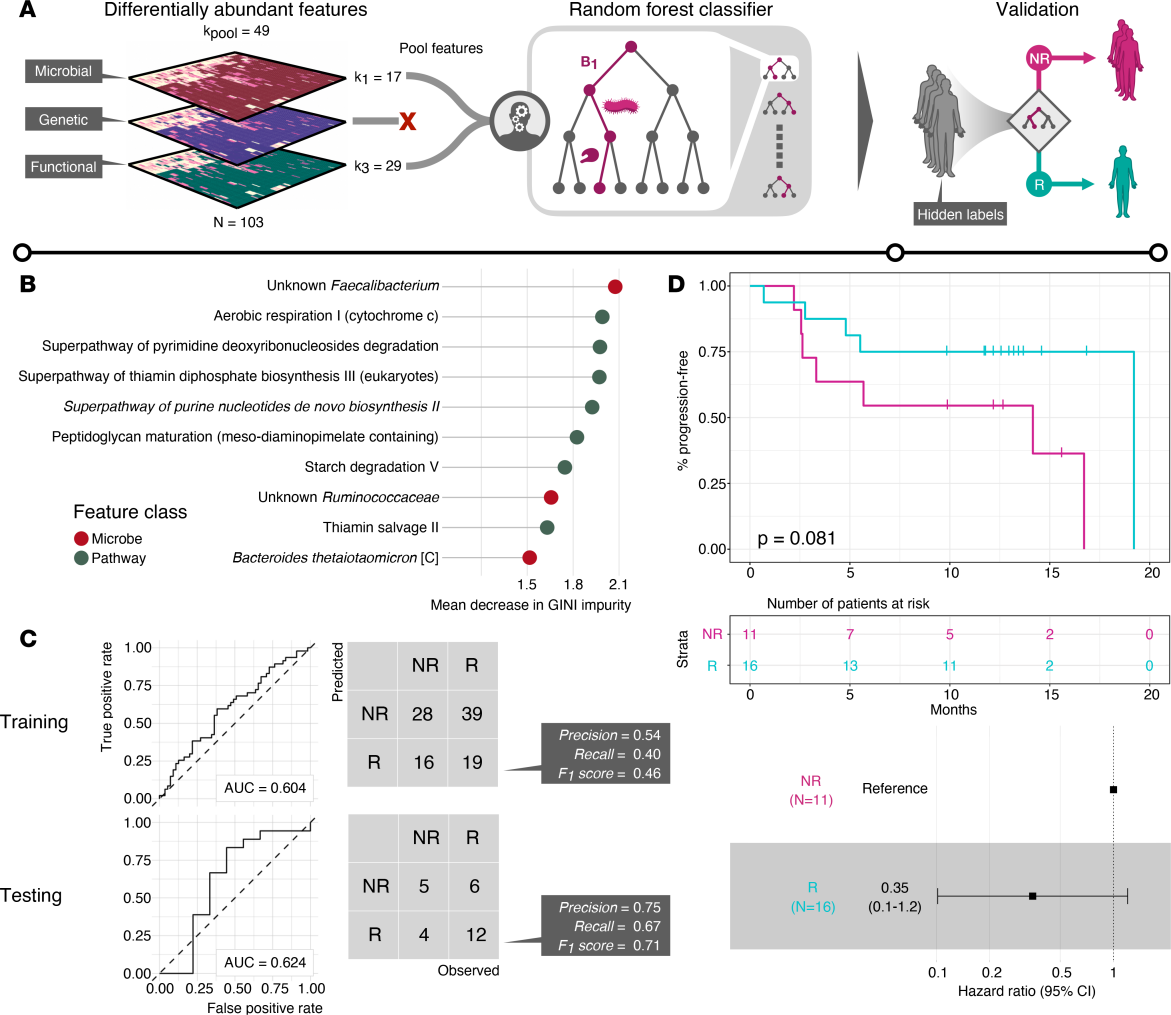
Microbial signatures in patients with melanoma are predictive of PFS in an independent cohort. (**A**) Construction of a random forest (RF) classifier for treatment response to ICI, based on fecal sequencing data. Differentially abundant features (*k_pool_* = 49) between R and NR patients (*N* = 103) were selected as input for training an RF classifier. The trained model was then used to predict treatment responses of patients in an independent cohort (*N* = 27). (**B**) Lollipop plot of the top 10 most important variables, evaluated according to the mean decrease in Gini impurity as determined by the RF classifier after model training. Features are color-coded according to species or pathway. (**C**) Performance characteristics of the RF classifier. Left: Receiver operating characteristic (ROC) curves on the training and test data. AUC, area under the curve. Right: Confusion matrices and prediction scores for the RF classifier on the training and test data. (**D**) Kaplan-Meier PFS estimates for the R and NR patients, as predicted by the RF classifier (*P* value using the log-rank test), with number of patients at risk for each 5-month interval and hazard ratio for the R group (calculated using Cox proportional hazards regression).

These results suggest that taxonomic as well as functional microbial features define a complex signature associated with response to ICI in MM. However, our findings also highlighted substantial heterogeneity across the studies questioning whether these features are actually predictive of response to ICI or are a spurious finding resulting from reducing the initial dimensionality until a separation between the groups was achieved. Therefore, we decided to validate this signature in an independent cohort. Specifically, we tested whether the feature set was predictive of response in the study by Peters et al. (18). We tackled this through a machine learning–based approach, where we used our differentially abundant species and pathways as selected feature inputs for a random forest (RF) classifier (Figure 4A). After training the RF classifier, we examined the most important features in the classification, evaluated by mean decrease in Gini impurity (Figure 4B). The most predictive feature overall was the abundance of an unknown *Faecalibacterium*, with the second most being the abundance of aerobic respiration (PWY-3781). Classification performance of the model on the training data was evaluated based on the receiver operating characteristic curve (ROC), reaching an area under the ROC curve (AUC) of 0.604 (95% CI: 0.511–0.709), indicating a modest yet nonrandom separation between R and NR (Figure 4C, left). Because Peters et al. did not classify patients into R versus NR explicitly (18), we categorized each patient as belonging to R or NR based on PFS less than or greater than 6 months, respectively, consistent with the other studies. The prespecified RF classifier achieved a classification performance on the test data consistent with the training sets (Figure 4C, right, AUC = 0.624, 95% CI: 0.348–0.899). When we performed unsupervised hierarchical clustering based on the features used in the RF classifiers to examine patients in the validation data, we observed a separation between the R and NR classes (Supplemental Figure 8). This is strongly suggestive that the here-reported signature incorporating taxonomic and functional features of the gut microbiome represents a modest yet predictive factor of response to ICI in MM.

Next, we investigated whether grouping of patients into R versus NR according to the RF classifier was prognostic in the validation data in terms of PFS. Considering the fact that only 27 baseline samples were present in the validation data set (*N* = 16 responders), we sought to determine the statistical power of a log-rank test by performing an a priori minimum detectable effect (MDE) calculation and so quantify the required hazard ratio between the R and NR groups at a 5% significance level. The MDE for the hazard ratio was 0.17 (Methods). We observed that categorization of patients into R according to the RF classifier resulted in a hazard ratio of 0.35 for PFS, as shown in Figure 4D, bottom. Kaplan-Meier survival estimates managed to separate both classes, albeit not enough to reach significance (*P* = 0.081, log-rank test, Figure 4D). This was expected given we previously determined that the log-rank test was underpowered to detect significance at 5% confidence level for hazard ratio estimates greater than 0.16. This finding extends the previous result and suggests that the here-reported signature is also quantitatively correlated to the duration of response to ICI in an independent cohort of patients with MM.

## Discussion

In this meta-analysis, we aimed to understand which features of the microbiome consistently correlate with response to ICI across studies in patients with MM. In addition, we investigated several additional features as well as mapped out their likely biological role in mediating the clinical benefits. Global microbiome structure does not seem to cluster based on response phenotype alone, as evident by the poor separation between classes in the MDS plots. This could owe to other factors that correlate with microbial composition in a more direct way, such as genetics, diet, geographical location, and choice of protocols for bacterial lysis and DNA extraction. It is worth noting that the lack of global separation may also be due to inherent biases caused by the experimental design, including patient preselection. For example, patient microbiomes might be initially characterized using 16S rRNA gene sequencing (16S-Seq), and then a subset of these with interesting microbial patterns may then be further analyzed in detail using whole-genome sequencing, which is more costly. The advantage of a meta-analysis is to identify factors independent of biases introduced by specific choices in the experimental design of a given study and are therefore more likely to be inherent to response and whole-body immunity. We also did not observe higher microbial community diversity in the responder group. In fact, the importance of community diversity in ICI outcomes remains to be determined. Although higher community diversity has generally been associated with a microbiome belonging to a healthy state across multiple diseases (21–23), only the Gopalakrishnan et al. study (15), as well as the 16S-Seq data from the Peters et al. study (18), have reported higher diversity in responders to ICI. A previous meta-analysis of microbial data sets found that reduced alpha-diversity is only characteristic of very few diseases (24), and our study agrees that this is also the case in MM patients’ response to ICI. When analyzing community structure through co-occurrence networks, we found several genera serving as network hubs. However, the implications of these in cancer or immunity has yet to be determined, with the exception of *Lachnoclostridium*, which has recently been found to be a marker for the progression of colorectal cancer (25). Targeting the large hubs in the nonresponder network might serve as a way to alter the microbiome composition in these patients, although it is important to note that co-occurrence of 2 microbes does not imply that the microbes share any dependencies for growth.

At the microbial composition level, we reidentified several beneficial taxa that had been previously described as enriched in responders of ICI in melanoma. We stress that the methods chosen for significance testing are not statistically rigorous due to no adjustment for multiple testing being used; instead we used them simply to extract features that differed between R and NR. The differentially abundant taxa in R included species in the *Faecalibacterium* genus and Ruminococcaceae family, both of which were associated with R in several of the studies included in this analysis. Interestingly, by pooling data and increasing statistical power, we discovered additional taxa in the context of response to ICI. This includes *Barnesiella intestinihominis*, which has been shown to control the efficacy of the immunomodulatory chemotherapeutic cyclophosphamide in mice by inducing IFN-γ production by γδ T cells at the site of the tumor (26). It can therefore be conjectured that this mechanism could be conserved in humans also. We also identified *Ruminococcus bicirculans* as being enriched in R. *Ruminococcus bicirculans* was shown to harbor a specialized ability to degrade certain plant hemicelluloses (27). This is an important property for shaping and maintaining a functional microbiome composition through, for instance, supplying molecular hydrogen to neighboring Archaea (28). Surprisingly, *Bacteroides thetaiotaomicron* was found to be enriched in the NR group among the pooled MM patients, contrary to the findings of Peters et al., where it was enriched in R (18). This might be due to study-specific differences in microbiome composition, and further studies are needed to understand the effect of *Bacteroides thetaiotaomicron* on ICI response. *Bacteroides thetaiotaomicron* has recently been identified as having cancer-preventative activities, by metabolizing compounds found in cruciferous vegetables into chemopreventative isothiocyanates (29). Additionally, we identified the taxon *Adlercreutzia equolifaciens* as being enriched in NR in our training data, whereas it was enriched in R in the Peters et al. data set (Supplemental Figure 8). *Adlercreutzia equolifaciens* is a producer of equol (30), a polyphenolic compound able to bind to the human estrogen receptor–β, although how and whether this potentially relates to negative stimulation of immunity remain to be understood.

At the functional level, several of the processes enriched in R were centered on B vitamin metabolism, more specifically that of thiamine (vitamin B_1_) and cobalamin (vitamin B_12_). Data from the Peters et al. study had indicated the riboflavin (vitamin B_2_), pantothenate (vitamin B_5_), pyroxidal 5-phosphate (vitamin B_6_), and folate (vitamin B_9_) biosynthesis pathways as being predominantly present in NR (18), whereas a prior study investigating links between the gut microbiota and incidences of ICI-induced colitis events identified riboflavin, pantothenate, and thiamine as enriched in patients with low risk for developing colitis (31). Specific studies examining direct links between B vitamin supplementation and immune system modulation have also been conducted (32–34). These include a randomized controlled trial examining vitamin supplementation in patients with HIV, of which vitamin B_2_ and B_12_ were among the included vitamins. The vitamin-supplemented group had significantly higher CD8^+^ and CD4^+^ T cell counts, as well as lower disease progression (34). Because we identified 3 independent thiamine metabolic pathways, we hypothesize that supplementation of either the metabolite itself, or thiamine-producing bacteria, might be an interesting avenue to explore as an adjuvant in conjunction with current ICI regimens. However, the exact role of microbially produced B vitamins in the context of ICI will have to be further evaluated in future studies, as well as whether these findings represent a causal process for increased immune system stimulation. We also found several catabolic pathways, e.g., starch, glycoside, and nucleotides, enriched in R. These could potentially indicate features of a healthy microbiota, whereby microbial catabolism of these compounds allows the host to absorb and utilize microbially derived products. This stands in contrast with the aerobic respiration and nucleotide biosynthesis pathways enriched in NR, which are features of noncommensality and increased growth, respectively. We confirmed this by identifying that aerobic respiration seems to in part be driven by the *Campylobacter* and *Citrobacter* genera, both of which are opportunistic pathogens. In summary, analysis of the functional content of the gut microbiota is suggestive that vitamin B metabolism may be mechanistically implicated in ICI response, whereas a healthy microbiota could be a prerequisite for response.

The differentially abundant microbial features constituted a signature of response to ICI in MM. We constructed an RF classifier based on this signature in order to examine whether we could predict patient response. We found that *Faecalibacterium* was significantly more abundant in R (mean log_2_ fold change of 2.54) as well as being the most important predictor in the classifier, which further validates its importance in ICI treatment outcomes. Notably, 7 out of the 10 most important features consisted of functional pathways, which could imply that function might be a more generalizable feature of response compared with individual microbes. The fact that microbiome-associated changes correlated with response in an independent cohort is strongly suggestive that this signature could be generalized and underscores its contribution to the response to ICI in MM. However, the performance of the model needs to be increased before microbiome associated changes can be used as a predictor for response to ICI across distinct patient populations. Performance could potentially be increased by including additional layers of omics data, such as metatranscriptomics or metaproteomics, allowing for a more functional snapshot of the biological processes that are being actively expressed compared with MGS. Furthermore, it would be of interest to generate predictive models that incorporate additional clinical parameters (age, sex, body mass index, serum lactate dehydrogenase levels), data from patient tumors (whole-genome sequencing, chromatin accessibility assays, RNA-Seq), and immune system state (immune profiling panels, TCR-Seq), along with fecal metagenomics data.

In conclusion, this meta-analysis provides several microbial features predictive of response that are generalizable across studies. These features will have to be experimentally validated in future studies to verify their therapeutic or predictive potential.

## Methods

### Literature review.

We began by performing a literature search on PubMed/MeSH for any published articles relating to immunotherapy and the gut microbiome in humans using the following query (performed in February 2019): “*(((((“journal article”[Publication Type]) AND (“immunotherapy”[MeSH Major Topic])) AND (“microbiota”[MeSH Terms])) AND (humans[MeSH Terms])) NOT (“review”[Publication Type]))*”

This resulted in 38 records, which we screened and excluded all studies that did not contain human fecal MGS data from melanoma patients undergoing checkpoint immunotherapy. In total, we obtained 3 eligible studies from PubMed, Gopalakrishnan et al. (15), Matson et al. (16), and Routy et al. (20). Additionally, we identified the Frankel et al. study from the references of a perspective piece by Gopalakrishnan et al. (35). We repeated the search on PubMed in December 2019 using the same query, which allowed us to identify and include another eligible study by Peters et al. (18). Finally, after initial analysis (Supplemental Figure 1–4, Supplemental Figure 6), we restricted the scope of our studies to melanoma patients, thereby discarding the Routy et al. data set, which only included RCC and NSCLC patients. To our knowledge, no other studies relating checkpoint immunotherapy outcomes to fecal MGS data have been performed since the Peters et al. study. No protocol exists for the literature review.

### Data set and metadata collection.

MGS data from each study were obtained from the European Nucleotide Archive under the accession numbers PRJEB22893, PRJNA399742 (15), PRJNA397906 (15), PRJEB22863 (15), and PRJNA541981 (15). We excluded any samples taken after commencing ICI therapy, non-MGS samples, and nonfecal samples. Patient metadata from each study were obtained through the main manuscript for the Frankel et al. data; the study GitHub repository (https://github.com/cribioinfo/sci2017_analysis) for the Matson et al. data; NCBI BioProject (https://www.ncbi.nlm.nih.gov/bioproject) with accession ID PRJEB22863 for the Routy et al. data; and European Genome-phenome Archive with accession ID EGAS00001002698 and access granted from the study authors for the Gopalakrishnan et al. data set. All scripts, files, and data used for the analysis are publicly available on GitHub at https://github.com/angelolimeta/Gut-microbiome-immunotherapy Processing steps utilizing command line tools were performed in a reproducible Conda environment (Anaconda, Version 2-2.4.0, https://www.anaconda.com) with the Snakemake workflow engine (36) on a computational cluster.

### Patient stratification.

In order to avoid bias from differing assignments of treatment response criteria, we consistently classified patients into responder and nonresponder groups through the RECIST 1.1 (19). Patients with complete or partial response, according to RECIST 1.1 criteria at first evaluation, were classified as responders, whereas patients with stable or progressive disease were classified as nonresponders.

### Summary measures.

The principal summary measures used throughout the analysis were log-normalized relative abundances of microbes and pathways in each sample.

### Microbial composition analysis.

Raw FASTQ reads were quality filtered using fastp (37) with default parameters. In order to profile the microbial composition in each sample, we opted for a phylogenetic marker gene alignment–based approach using mOTUs2 (38) with default parameters. The resulting output from mOTUs2 after merging all samples is a tab-separated file containing, for each sample, counts for the number of reads that could be mapped to a given phylogenetic marker gene. Each marker gene is a single-copy gene acting as a marker for a given operational taxonomic unit (OTU), i.e., a microbe profiled at a specific phylogenetic resolution. For downstream analyses we also downloaded the phylogenetic tree file for all the OTUs included in mOTUs2. The tab-separated output file from mOTUs, phylogenetic tree file, and sample metadata were loaded into R and packaged into a phyloseq (39) object for ease of analysis. In order to adjust for sequencing depth across samples, raw counts for each OTU were converted into relative abundance values by dividing by the total amount of counts for each sample. For dimensionality reduction and visualization purposes, the relative abundances were also log-normalized. A small value of 10^–8^ was added to the relative abundances prior to normalization to avoid taking the logarithm of 0. Microbes not present in at least 5 samples were discarded from the normalized data set.

Global differences in microbial composition between patients were visualized using weighted UniFrac distance-based dimensionality reduction techniques, through incorporation of the phylogenetic tree data from mOTUs2. UniFrac variants of the MDS and t-distributed stochastic neighbor embedding (https://github.com/opisthokonta/tsnemicrobiota) algorithms were both used. Phylogenetic alpha-diversity indexes (Shannon, Inverted Simpson, ACE, and Chao1) were calculated for each sample, based on the identified OTUs. Differences in alpha-diversity between R and NR were assessed by applying 2-sided Wilcoxon’s rank-sum tests. Differentially abundant/present microbes between R and NR were extracted using 2 different methods. First, we performed 2-sided Wilcoxon’s rank-sum tests between R and NR on the log-normalized CoPM abundance for each microbe and considered any microbe with a *P* value (unadjusted for multiple testing) of less than 0.05 to be differentially abundant. Second, we ran through the raw microbial count data through the DESeq2 tool (40) and included treatment response (R and NR), antibody type (anti–PD-1, anti-CTLA-4, and combination therapy), and source study in the design matrix. Size factor estimation in DESeq2 was performed using the poscounts method. *P* values between R and NR obtained for each microbe were adjusted using the Benjamini-Hochberg procedure into *q* values, and all microbes with a *q* < 0.05 were considered differentially abundant.

### Co-occurrence network analysis.

Evaluating which microbes co-occur, i.e., their abundances are positively correlated, across a certain group of patients, e.g., R and NR, can yield insights into the network structure of the microbiome and uncover functional relationships between individual microbes. We performed co-occurrence analysis using the microbiomeSeq R package, for both the R and NR patient subsets. We defined pairs of microbes as co-occurring across samples if their Pearson’s correlation coefficient was above 0.3. *P* values obtained from pairwise correlations were adjusted using the Benjamini-Hochberg procedure.

### Functional profiling of the microbiome.

In order to profile the functional content, i.e., abundance of different pathways and genes/proteins, of the microbiota between samples, we used the HMP Unified Metabolic Analysis Network 2 (HUMAnN2) tool (41). HUMAnN2 was used in conjunction with the following databases: CHOCOPhlAn version 20, used for intermediate microbial profiling; UniRef50 gene clusters, used for quantifying genes/proteins; and MetaCyc in conjunction with the UniRef50 gene clusters, used for quantifying pathway abundances and coverages. In order to visualize global differences in the functional profile for each patient, we first utilized standard principal components analysis. In addition to this, we applied the manifold learning technique uniform manifold approximation and projection (UMAP) (42) on the data sets in order to capture nonlinear patterns in the data. UMAP was performed using the umap R package with default parameters (https://github.com/tkonopka/umap). Differential abundance tests between R and NR were performed using the 2-sided Wilcoxon’s rank-sum test, and all pathways with an unadjusted *P* < 0.05 were considered differentially abundant.

### Machine learning.

Differentially abundant microbes and pathways were pooled together and used to train an RF classifier with 100,000 trees (43). The RF classifier was exclusively trained on data from the melanoma studies, then used to predict response labels on fecal whole-genome sequencing data obtained from patients in the validation data set. Model performance on the training and testing data was evaluated using ROC curves, as well as contingency matrix metrics. Individual features in the trained classifier were ranked according to importance using the mean decrease in GINI metric.

Since the validation data set contained a limited number of participants (*N* = 27), we decided to perform an a priori MDE analysis to gauge how well our model needs to perform to achieve statistical significance. Our RF classifier classified each patient in this data set as belonging to R or NR, and we then performed a log-rank test comparing each patient group based on PFS. The MDE analysis tells us the minimum hazard ratio required to achieve significant differences in PFS (*P* > 0.05) for a given power level. We utilized the Freedman method (44) for calculating the critical hazard ratio:

  (Equation 1),
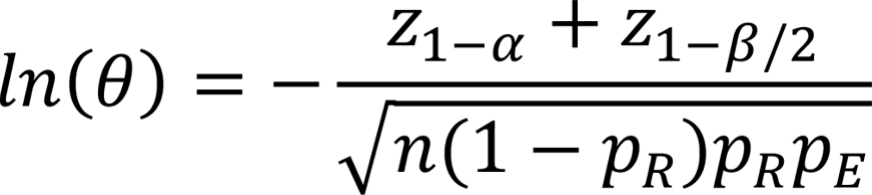


where *ln(θ)* is the log-hazard ratio, *z* is the probit function, *α* is the type I error, *β* is the type II error, *n* is the total number of patients, *p_R_* is the proportion of true responders (PFS > 6 months), and *p_E_* is the overall probability of progression occurring within the study period. From our data we obtained *n* = 27, *p_R_* = 0.66, *p_E_* = 0.44. Setting a type I error level of 0.05 and a type II error level of 0.2 yielded a critical log-hazard ratio of –1.79, resulting in a hazard ratio of 0.166.

### Availability of code.

All scripts used for the processing and analysis of data are available at https://github.com/angelolimeta/Gut-microbiome-immunotherapy

### Statistics.

Analyses of processed metagenomics data were conducted in the R software package (version 3.6.1 “Action of the Toes”; The R Project for Statistical Computing, http://www.R-project.org). Two-tailed *P* values less than 0.05 were considered statistically significant across all tests. Statistical comparisons of log-normalized microbial and pathway abundances, and microbial alpha-diversity indexes between groups, were performed using the 2-sided Wilcoxon’s rank-sum test. Fisher’s exact test was utilized for clustering comparisons of patients using differentially abundant features. When analyzing differences in survival between predicted patient groups, we utilized the log-rank test.

### Study approval.

Study approval was not required as all the patient data used in this study are anonymized and already exist in the public domain.

## Author contributions

AL, BJ, and JN conceived and designed the study. AL, BJ, and FG analyzed the data. AL, BJ, FG, and ML wrote the paper. All authors were involved in interpretation, editing, and discussion.

## Supplementary Material

Supplemental data

Trial reporting checklists

Supplemental Table 1

## Figures and Tables

**Table 1:**
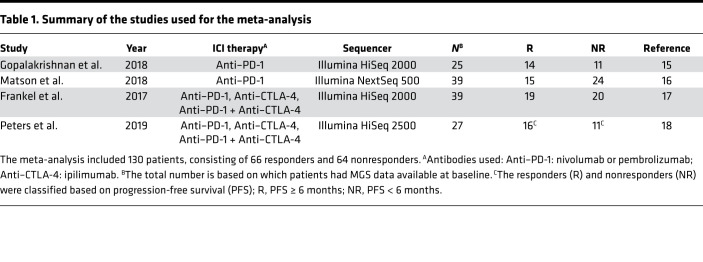
Summary of the studies used for the meta-analysis

## Version 1 12/03/2020

Electronic publication
