# Supplementary material for: Meta-analysis of the gut microbiota in predicting response to cancer immunotherapy in metastatic melanoma
Source: JCI Insight. 2020 Dec 3;5(23):e140940. doi: 10.1172/jci.insight.140940 (PMC7714408; doi:10.1172/jci.insight.140940)
Supplement: Supplemental data [file jciinsight-5-140940-s169.pdf]

Abbreviations

- **AUC:** area under the curve.
- **CD4+ T-cell:** T helper cell
- **CD8+ T-cell:** cytotoxic T-cell
- **CDK:** cyclin dependent kinase
- **CI:** confidence interval
- **CoPM:** copies per million
- **CTLA-4:** cytotoxic T-lymphocyte-associated antigen 4
- **CTX:** cyclophosphamide
- **ERβ:** human estrogen receptor beta
- **HLA:** human leukocyte antigen
- **ICI:** immune checkpoint inhibitor
- **IFN- γ:** interferon γ
- **MDE:** minimum detectable effect
- **MDS:** multi-dimensional scaling
- **MGS:** whole metagenome shotgun sequencing
- **MM:** metastatic melanoma
- **NR:** non-responder
- **NSCLC:** non-small cell lung cancer
- **PD-1:** programmed cell death 1 protein
- **PD-L1:** programmed death ligand 1
- **PFS:** progression-free survival
- **R:** responder
- **RCC:** renal cell carcinoma
- **RF:** random forest
- **ROC**: receiver operating characteristic curve
- **TPM:** transcripts per million

Supplemental Figures

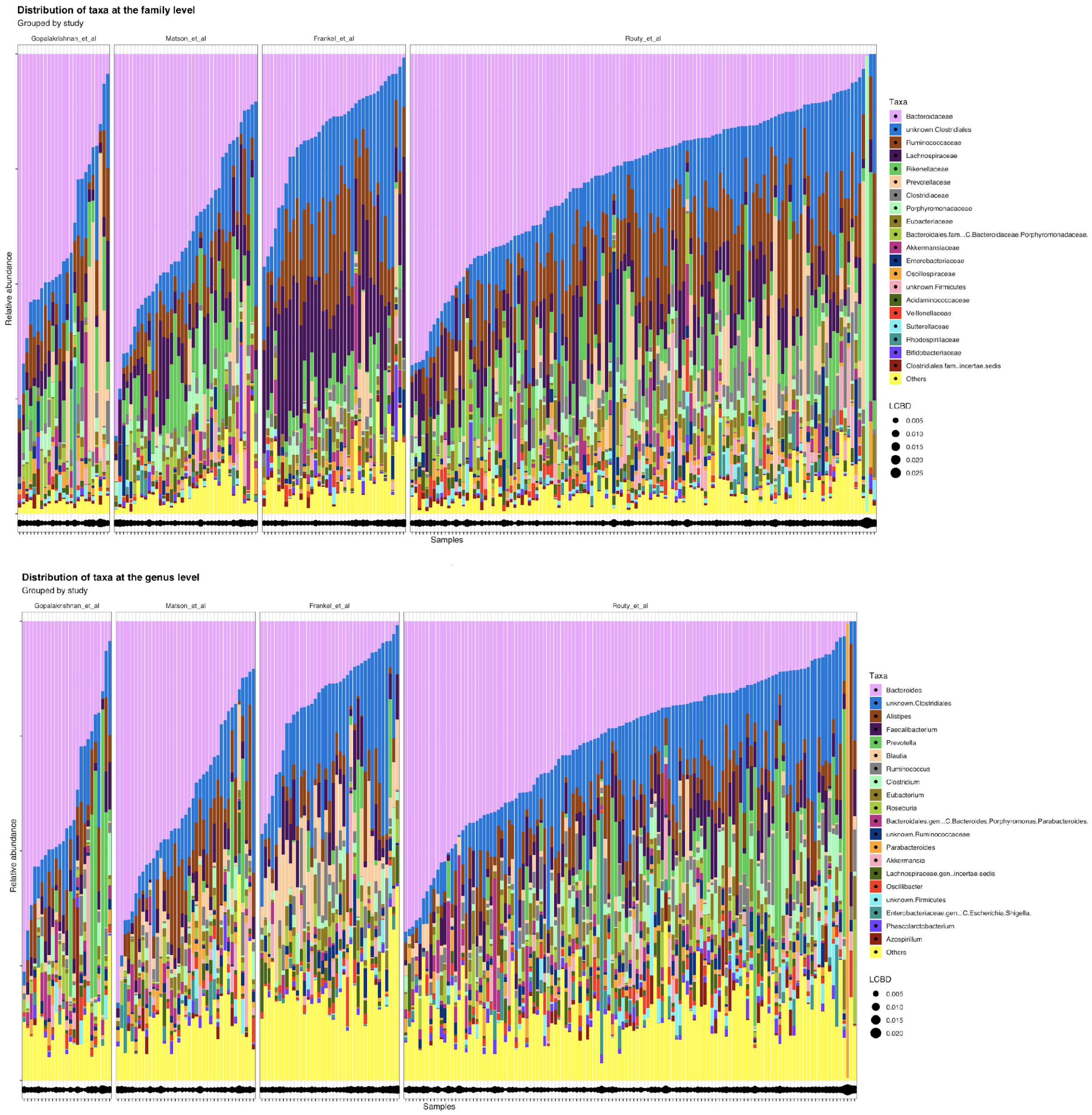

Fig. S1. Distribution of taxa for each of the four studies used for exploratory analysis. Top: Family-level. Bottom: Genus-level. Circle sizes in the bottom of the plot are proportional to the Local Contribution to Beta-Diversity (LCBD) for each study. Samples with large LCBD values differ the most from the remaining samples in the study.

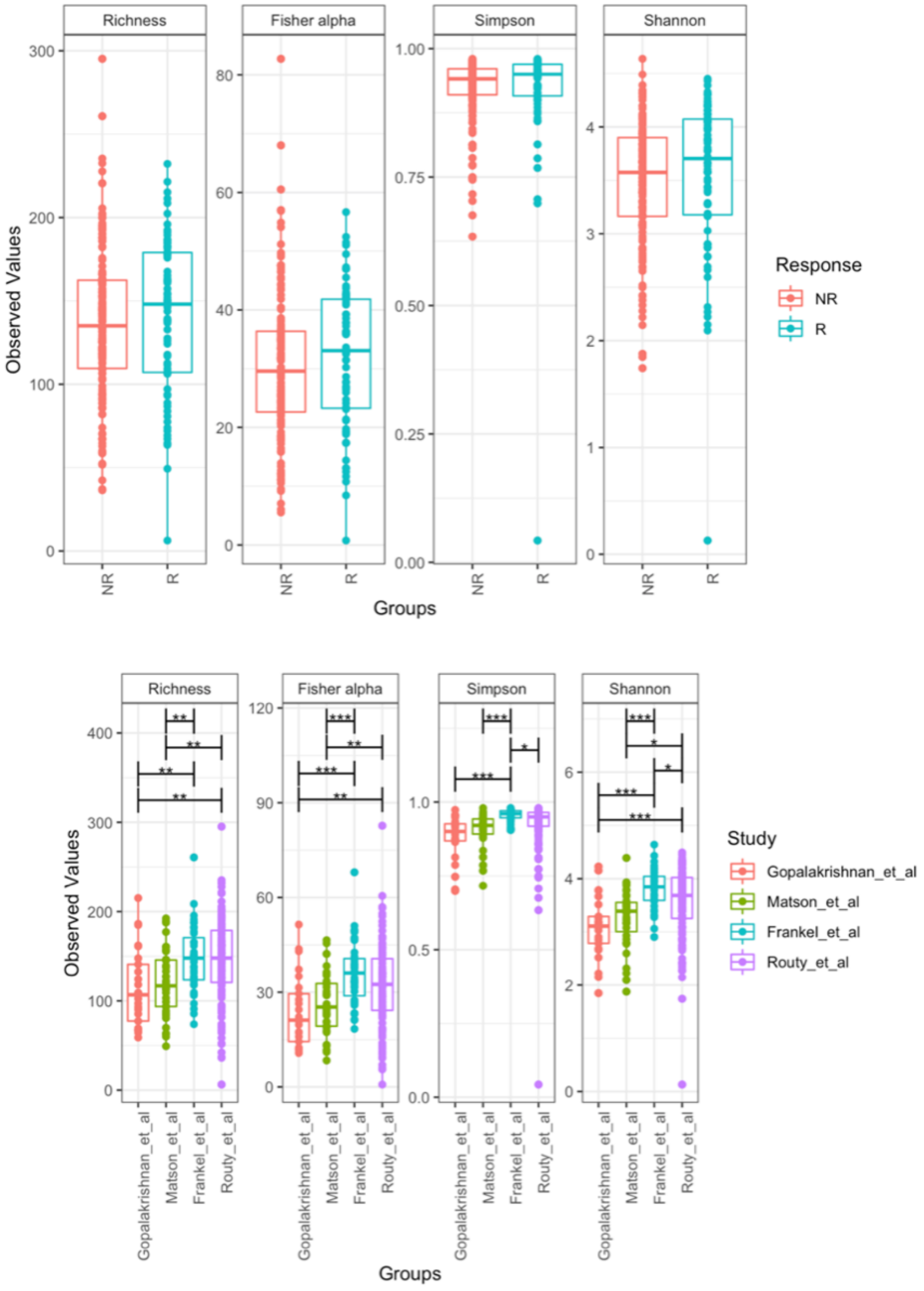

Fig. S2 Differences in Alpha diversity. Top: R *vs* NR. None of the alpha diversity indexes were significantly different between groups. Bottom: differences between studies. *$p < 0.05$, **$p < 0.01$, ***$p < 0.001$, evaluated using one-way ANOVA with pairwise comparisons.

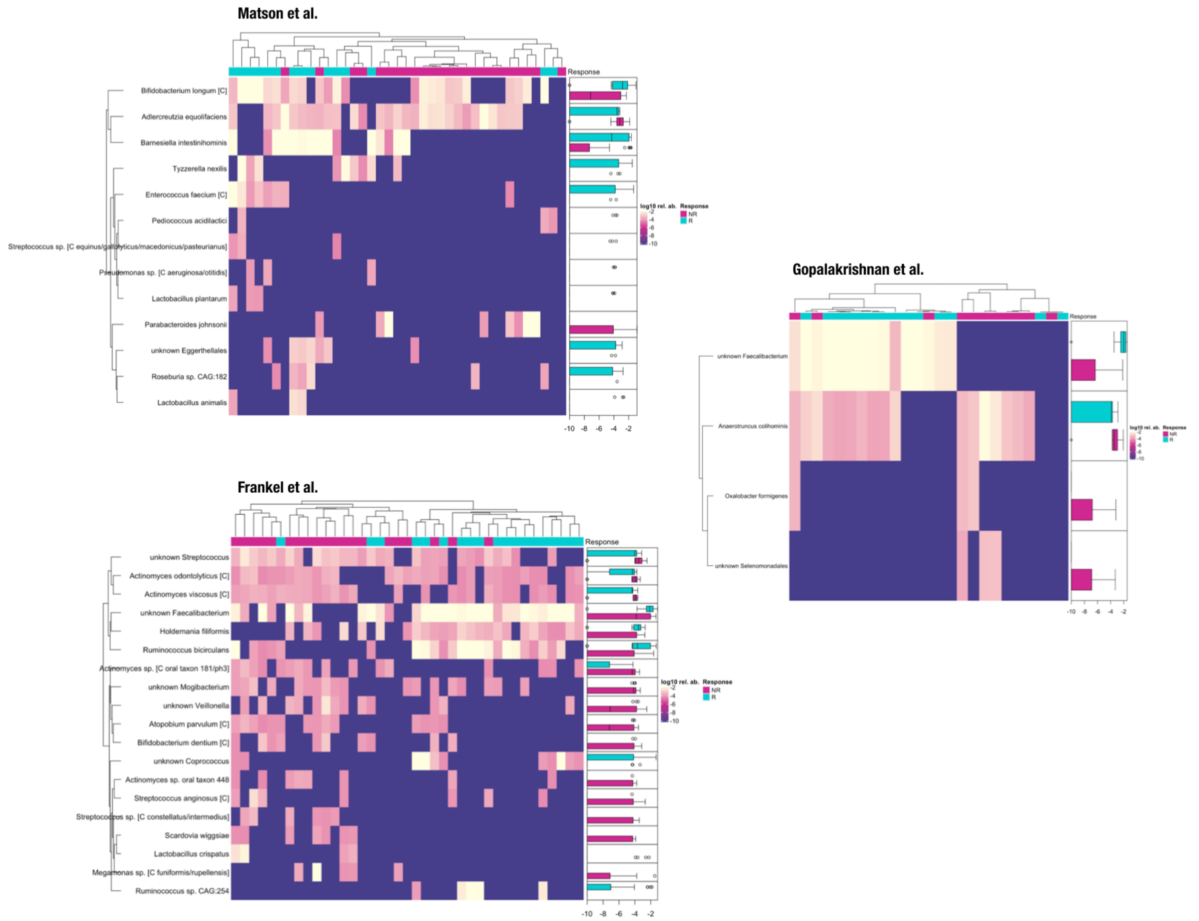

Fig. S3 Hierarchical clustering of differentially abundant microbial species between R and NR on a study by study basis. Species were identified as differentially abundant with $p < 0.05$ (unadjusted, Wilcoxon rank-sum test). Data from the Routy et al. data set is omitted as it did not produce any differentially abundant species between R and NR according to our response criteria.

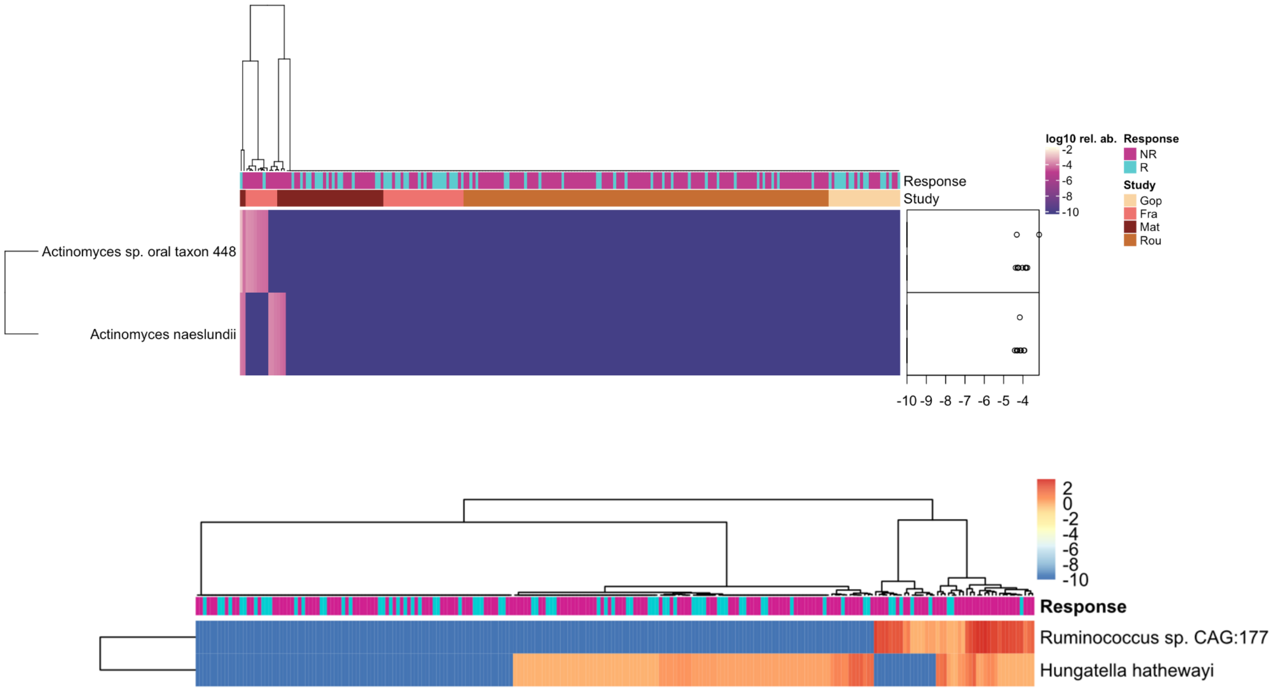

Fig. S4 Hierarchical clustering of differentially abundant microbial species between R and NR across all four studies. Top: Wilcoxon rank-sum test. Bottom: DESeq2.

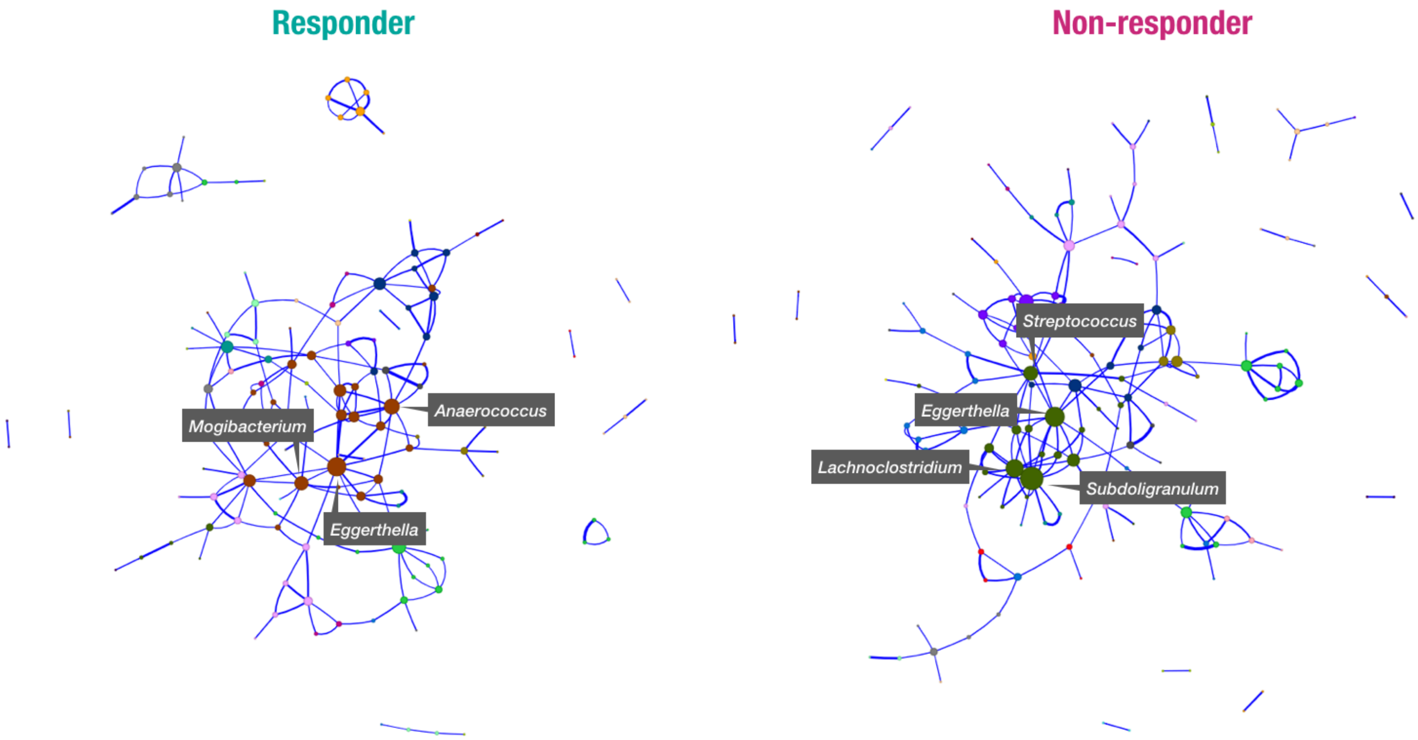

Fig. S5 Co-occurrence networks amongst responders and non-responders to immune checkpoint inhibition in metastatic melanoma. Microbial co-occurrence networks at the genus level in the R subset (top), and NR subset (bottom), for the three studies included in the exploratory analysis. Each node represents a microbe and each edge represents a co-occurrence with Pearson correlation coefficient (PCC) > 0.3. The size of each node is proportional to the number of surrounding edges, and the size of each edge is proportional to the value of the PCC. Genera with annotated names have 7 or more edges with PCC > 0.3.

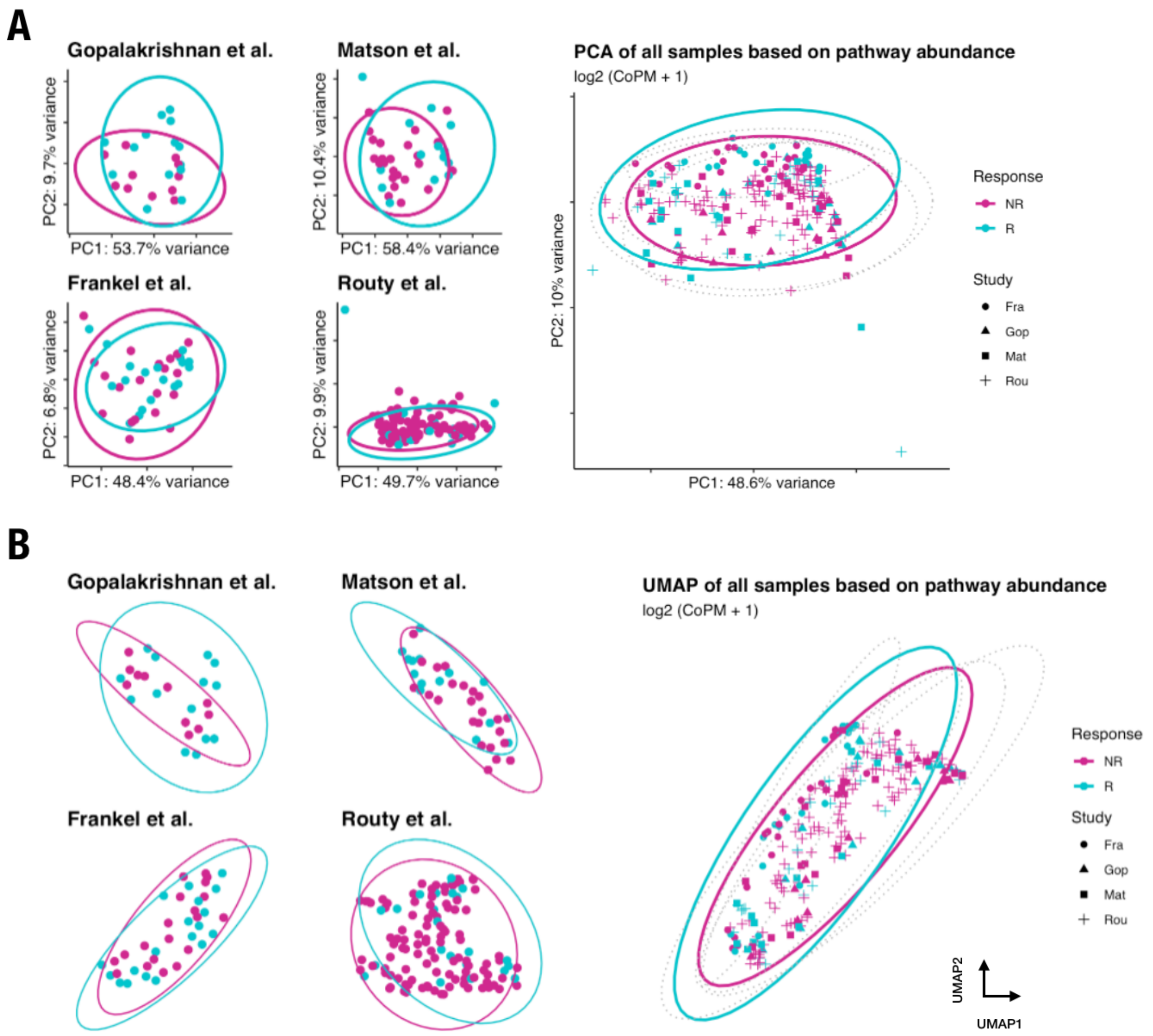

Figure S6. Dimensionality reduction analysis of the functional content in the gut microbiota between responders and non-responders to immune checkpoint inhibition. Input to the dimensionality reduction algorithms is log-normalized copies per million (CoPM, analogous to TPM for RNA-seq data) of MetaCyc pathways. (**A**) Principal component analysis (**B**) Uniform Manifold Approximation and Projection (UMAP) analysis.

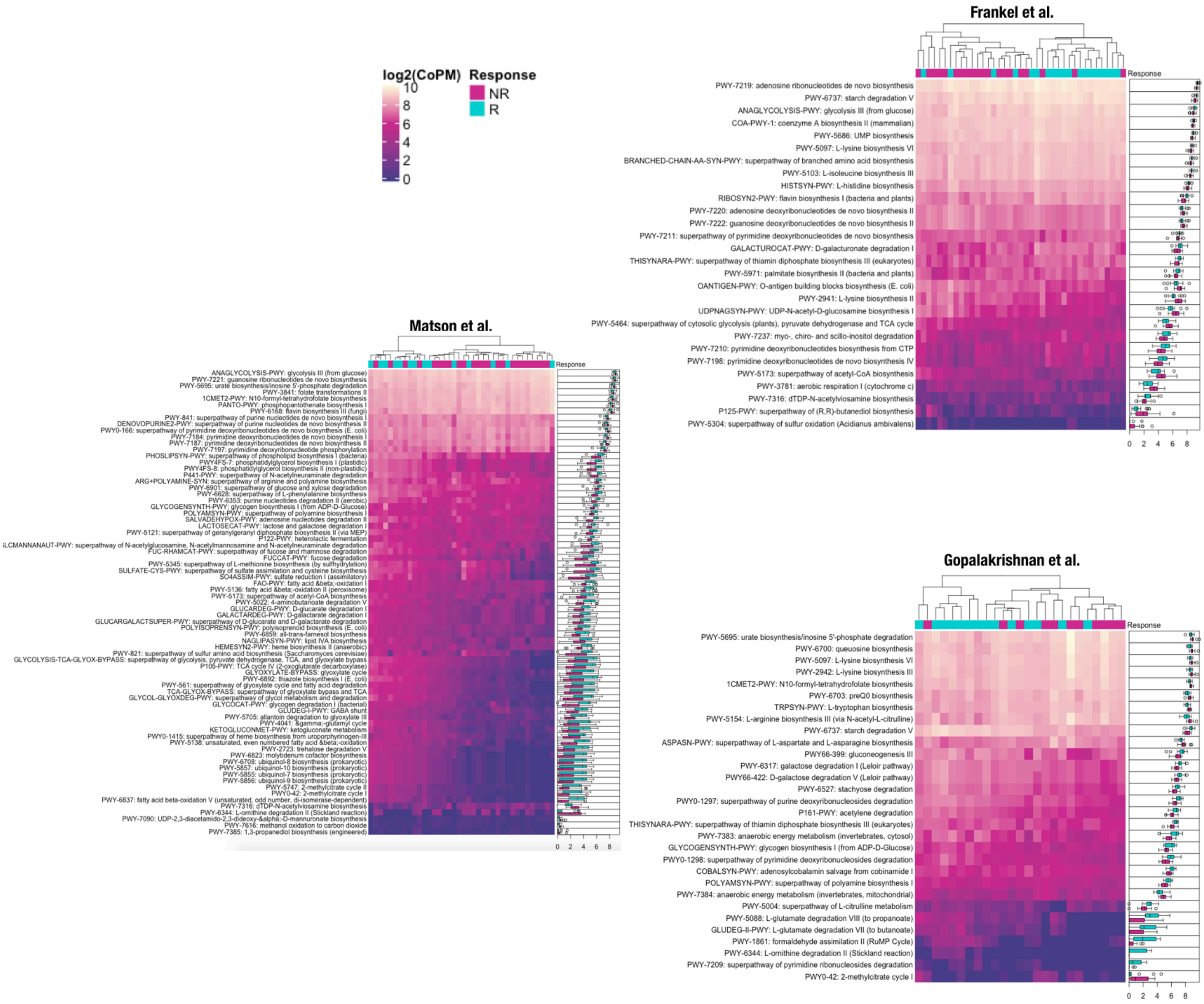

Fig. S7 Hierarchical clustering of differentially abundant MetaCyc pathways between R and NR on a study by study basis. log-normalized copies per million (CoPM, analogous to TPM for RNA-seq data) of differentially abundant, $p < 0.05$ (unadjusted, Wilcoxon rank-sum test) MetaCyc pathways present in the fecal microbiome of patients in each study. PWY-tags preceding each pathway name are unique IDs associated with each metabolic pathway on MetaCyc. Hierarchical clustering dendrogram for the pathways is omitted due to figure size constraints.

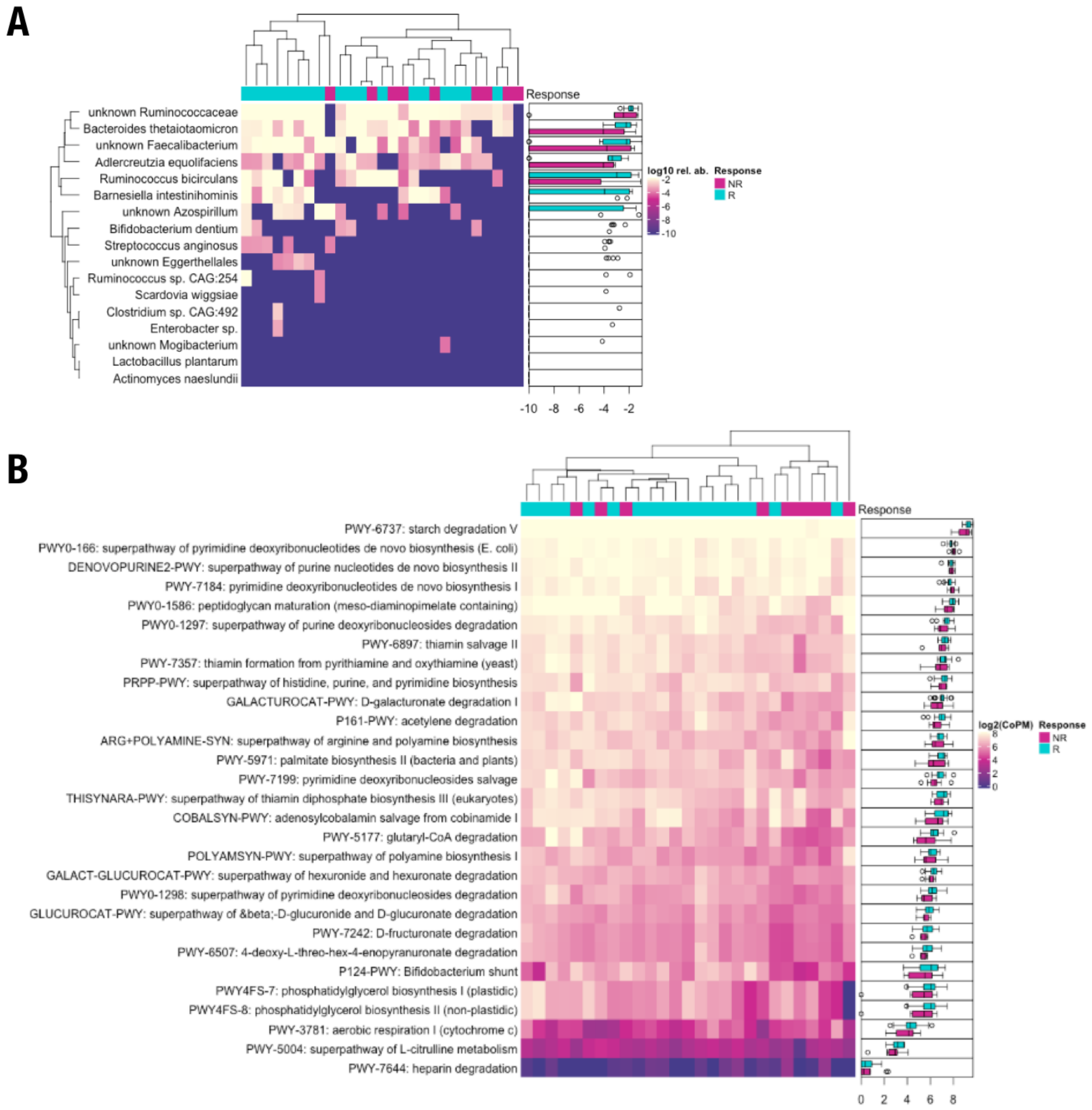

Figure S8. Hierarchical clustering of the input features of the random forest classifier in the validation data set. (**A**) Microbial features. (**B**) Functional features.
